# Craniofacial and dentoalveolar morphology in individuals with Prader–Willi syndrome: a case-control study

**DOI:** 10.1186/s13023-022-02222-y

**Published:** 2022-02-22

**Authors:** Gisela Vasconcelos, Jo S. Stenehjem, Stefan Axelsson, Ronnaug Saeves

**Affiliations:** 1 TAKO-Centre, National Resource Centre for Oral Health in Rare Medical Conditions, Lovisenberg Diaconal Hospital, Pb 4970 Nydalen, 0440 Oslo, Norway; 2 Department of Biostatistics, Oslo Centre for Biostatistics and Epidemiology, University of Oslo, Oslo, Norway; 3 Department of Research, Cancer Registry of Norway, Oslo, Norway

**Keywords:** Prader–Willi, Craniofacial, Dentoalveolar, Cephamoletric analysis

## Abstract

**Background:**

Prader–Willi syndrome (PWS) is a complex multisystem genetic disorder with distinct genetic and clinical features. Among other clinical symptoms, PWS is characterized by severe infantile hypotonia with feeding problems, childhood onset hyperphagia, obesity, scoliosis, short stature combined with growth hormone deficiency and developmental delay. PWS is associated with facial dysmorphology, orofacial dysfunction, oral abnormalities, low salivary flow and subsequent severe tooth wear. Little is known about the craniofacial growth direction or dental and skeletal relationships in individuals with PWS in different ages. The purpose of this study was to assess the craniofacial and dentoalveolar characteristics and to investigate the craniofacial growth direction separately in children, young adults and adults with PWS, using a cephalometric analysis of lateral cephalograms.

**Results:**

Lateral cephalograms of 42 individuals with a confirmed genetic diagnosis of PWS were analysed and divided into three groups according to their age: Children (< 12 years), young adults (12–20 years) and adults (> 20 years). Cephalometric variables were compared between PWS patients and controls by age and sex. Significant deviations and distinct craniofacial patterns were found in children, young adults and adults with PWS compared with the control group. Children showed retrognatic mandible with a skeletal class II relationship, posterior growth direction and longer anterior face height. The young adults had smaller cranial base angle, a skeletal class II pattern and a higher anterior lower face than the control group. Adults with PWS had a prognathic mandible, skeletal class III relationship with anterior growth direction, more retroclined lower incisors and proclined upper incisors than the controls. Similar results were found when comparing the three groups with PWS; the adults had a prognathic mandible, skeletal class III pattern and anterior growth direction. Children had a retropositioned mandibula, skeletal class II relationship and posterior growth direction.

**Conclusion:**

This study may contribute to a better understanding of the craniofacial growth pattern in children, young adults and adults with PWS and may have a clinical importance when planning dental treatment, such as prosthodontics and/or orthodontics.

## Background

Prader–Willi syndrome (PWS) is a complex multisystem genetic disorder affecting both genders equally. The prevalence is estimated to be between 1:25.000 and 1:52.000 [1–3]*.* PWS results from the lack of expression of the genes in the region q11–q13 on chromosome 15 [4]. A deletion of this area in the paternally inherited chromosome 15 is the most common cause (65–70%) [4, 5]. Another reason can be a maternal uniparental disomy (mUPD), where two copies of the maternal chromosome 15 are inherited but no paternal copy is present (20–30%). Less frequently, unbalanced translocations or imprinting defects can be present [4, 5].

PWS is characterized by severe infantile hypotonia with feeding problems, childhood onset hyperphagia, obesity, scoliosis, short stature associated with growth hormone (GH) deficiency and developmental delay [6, 7]. Facial dysmorphology include narrow bifrontal diameter, almond-shaped eyes, thin upper lip, down-turned corners of the mouth and small mouth in the infancy [7]. Dolichocephalic head shape is common [8, 9].

Saeves et al. carried out an interdisciplinary research on the oral characteristics of PWS including orofacial dysfunction, tooth wear and salivary flow rates [10–12]. Mild-to-severe oral motor dysfunction such as challenges with tongue, cheeks or facial mobility were more common in the PWS group than in the control group. Other orofacial dysfunctions included habits of grinding teeth during the day and breathing problems such as snoring during sleep [10]. Sleep-disorders, including obstructive, central, and mixed sleep apnea symptoms, were present in many individuals with PWS. In children, adeno-tonsillectomy is the first choice for treatment for obstructive sleep apnea. Additional therapies such as Continuous Positive Airway Pressure (CPAP) may be needed [13, 14].

It has been shown that low salivary flow rate and severe tooth wear are common findings in PWS, and that there seems to be an increased risk for tooth wear with reduced salivary secretion [11, 15]. In PWS, the prevalence of gastro-oesophageal reflux seems to be high and strongly associated with tooth wear [12].

There is little information about craniofacial features in PWS in the literature. Most publications on craniofacial characteristics in PWS are case reports [16, 17]. Only three scientific publications are, to our knowledge, based on larger number of individuals [18–20]. It seems that there is a general agreement in these studies that the skeletal structures in PWS is smaller than average in the general population, and it is not clear how craniofacial growth direction, dental and craniofacial relationships are at different ages in individuals with PWS.

The aim of this study was to describe the craniofacial and dentoalveolar characteristics and to investigate the growth directions separately in three age groups of PWS; children, young adults and adults, and to compare these groups with each other and with healthy controls. This description is part of the multidisciplinary investigation with the same individuals with PWS initiated in 2010 of the oral aspects of PWS referred above [10–12, 21].

## Material and methods

### Ethical approval

The study protocol was approved by the Regional Committee for Medical Research Ethics (reference number: REK. 1.2006.14) and The Norwegian Data Inspectorate. Informed consent was obtained from all participants. When participants were under 18 years of age or were adults who had a guardian, informed consent was also acquired from the parents or guardian.

### Study population and control group

From an initial group of 50 individuals, lateral cephalograms of 42 individuals with a confirmed genetic diagnosis of PWS were included in this study. The lateral cephalograms of the remaining eight individuals were not included in the study due to poor quality of the radiograph or because the permanent incisors were not erupted. The selected participants were then divided into three groups according to their age: Children (< 12 years), young adults (12–20 years) and adults (> 20 years) (Table 1).

**Table 1:** Characteristics of the study population

	PWS < 12 years (n = 12)	PWS 12–20 years (n = 10)	PWS > 20 years (n = 20)	Control < 12 years (n = 66)	Control 12–20 years (n = 69)	Control > 20 years (n = 54)
Age, mean years (SD)	8.8 (± 2.1)	15.9 (± 2.3)	26.9 (± 6.8)	8.0 (± 1.4)	13.5 (± 2.0)	33.3 (± 1.5)
No. of males/females	5/7	6/4	11/9	19/47	15/54	16/38
Medication						
No. of GH users	11	9	17	0	0	0
GH treatment duration, mean months (SD)	56.2 (± 21.8)	114.7 (± 51.3)	67.1 (± 54.0)			

These lateral cephalograms from individuals in the three age groups were compared, by sex, with lateral cephalograms of a control group obtained from “The University of Oslo Craniofacial Growth Archives” [22].

All participants in the control group were without known or suspected disease. They represent a random selection of skeletal types and malocclusions at the various age levels in the general population (Table 1).

The lateral cephalograms were transferred to digital cephalometric analysis and examined by one of the authors (GV) (Facad Orthodontic Tracing Software, Ilexis AB, Linköping, Sweden).

### Data analysis

Before analysing the data in the study, thirteen lateral cephalograms from the PWS group were randomly chosen for tracing and digitalisation on two separate occasions more than three weeks apart in order to estimate systematic measurements errors. When comparing means of the cephalometric variables in the PWS group and control group, we used an independent sample t-test. When comparing means within a group, a paired t-test was used. Test–retest reliability was evaluated by using the intra-class correlation coefficient (ICC). All tests were two-sided with a level of significance of 0.05. Data from all variables were analysed using the statistical package SPSS© Base 22.0 (SPSS Inc., Chicago, IL, USA).

**Fig. 1:**
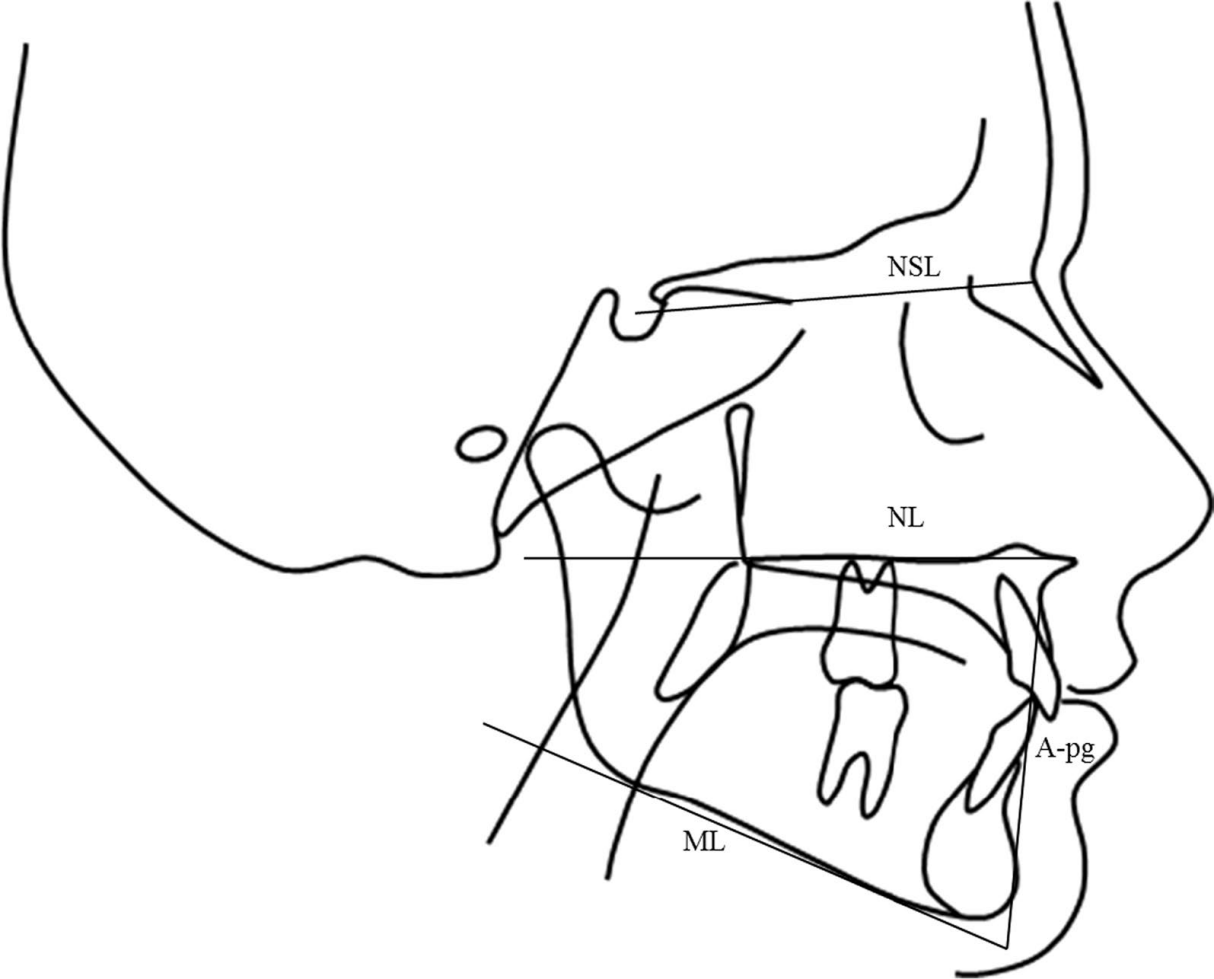
Landmarks and reference lines for linear and angular measurements in the lateral cephalogram. *NSL* nasion-sella line, *NL* maxillary line, *ML* mandibular line, *A-pg* line from subspinale to pogonion

The reference points and lines used were in accordance by Björk [23] (Fig. 1 and Table 2).

**Table 2:** Landmarks and reference lines for linear and angular measurements in the lateral cephalogram

Basal sagittal		
n-s-ba	Cranial base (nasion-sella-basion)	Degree
s-n-A	This angle represents the relative anteroposterior position of the maxilla to the cranial base	Degree
s-n-B	This angle represents the relative anteroposterior position of the mandible to the cranial base	Degree
A-n-B	This angle represents the relative anteroposterior position of the maxilla to the mandible and can be used to determine skeletal class relationship	Degree
A ḻ n-pg	Perpendicular distance between point A and line nasion-pogonion	mm
NL/NSL	The angle formed between the Maxillary Line (NL) and nasion-sella line	Degree
ML/NSL	The angle formed between the Mandibular Line (ML) and nasion-sella line	Degree
ML/NL	The angle formed between the Maxillary Line and Mandibular Line	Degree
FA/n-ba	Degree of convexity of the face	Degree
n-sp	Line between nasion and spina	mm
sp-gn	Line between spina and gnathion	mm
N-sp/sp-gn	Relationship between upper and lower face	%
Dental		
Ii to A-pg	Distance from the incisal point of the mandibular incisors to the line A-pg	mm
Ili/A-pg	Angle formed between line A-pg and the axis of the mandibular incisors	Degree
Ili/ML	The angle between the mandibular plane and the axis of the mandibular incisors	Degree
Is to A-pg	Distance from the incisal point of the maxillary incisors to the line A-pg	mm
Ils/A-pg	The angle between line A-pg and the axis of the maxillary incisors	Degree

## Results

The evaluation of systematic measurements errors in the cephalograms showed an acceptable test–retest reliability, yielding ICCs between 0.90 and 0.99 (*p* < 0.05).

### Comparison between the PWS and control groups (Tables 3, 4, 5)

**Table 3:** Cephalometric measurements for PWS individuals and control group under 12 years of age

Measurement		PWS < 12 years (n = 12)		Control < 12 years (n = 47)		Difference	t-test *p* value
		Mean	SD	Mean	SD		
Basal sagittal							
n-s-ba	Degree	129.2	7.0	131.3	3.2	− 2.1	0.14
s-n-A	Degree	81.1	5.3	81.1	2.8	0	0.98
s-n-B	Degree	76.1	5.5	78.1	2.8	− 2.0	0.08
A-n-B	Degree	5.0	2.4	3.1	1.9	+ 1.9	0.003
A ḻ n-pg	mm	4.3	2.2	2.1	1.8	+ 2.2	0.001
Basal vertical							
NL/NSL	Degree	11.6	4.5	7.2	2.9	+ 4.4	0.006
ML/NSL	Degree	35.9	7.4	31.3	3.7	+ 4.6	0.003
ML/NL	Degree	24.3	7.6	24.1	3.7	+ 0.2	0.87
FA/n-ba	Degree	88.2	5.4	92.6	3.2	− 4.4	0.001
Anterior face height							
n-sp	mm	46.0	4.3	41.9	2.9	+ 4.1	0.007
sp-gn	mm	57.7	6.1	53.6	3.6	+ 4.1	0.003
n-sp/sp-gn	%	80.1	6.6	78.5	6.2	+ 1.6	0.42
Dental							
Ii to A-pg	mm	1.5	2.3	0.6	1.4	+ 0.9	0.10
ILi/A-pg	Degree	20.7	4.1	18.4	5.8	+ 2.3	0.24
ILi/ML	Degree	93.6	5.4	91.3	6.0	+ 2.3	0.28
Is to A-pg	mm	4.5	3.5	3.6	1.9	+ 0.9	0.28
ILs/A-pg	Degree	26.1	8.4	23.4	6.1	+ 2.7	0.24
ILs/NSL	Degree	96.3	7.7	99.2	6.9	− 2.9	0.24
ILi/ILs	Degree	133.2	9.4	138.2	10.4	− 5.0	0.17

Basal sagittal—*n-s-ba*: cranial base inclination, *s-n-A*: maxillary prognathism, *s-n-B*: mandibular prognathism, *A-n-B*: relative prognathism, *A ḻ n-pg*: facial convexity

Basal vertical—*NL/NSL*: maxillary inclination, *ML/NSL*: mandibular inclination, *ML/NL*: mand./max. inclination, *FA/n-ba*: facial axis

Anterior face height—*n-sp*: upper anterior face height, *sp-gn*: lower anterior face height, *n-sp/sp-gn*: ratio

Dental—*Ii to A-pg*: lower incisor protrusion, *ILi/A-pg*: lower incisor inclination to A-pg, *ILi/ML*: lower incisor inclination to ML, *Is to A-pg*: upper incisor protrusion, *ILs/A-pg*: upper incisor inclination to A-pg, *ILs/NSL*: upper incisor inclination to NSL, *ILi/ILs*: interincisor angle

**Table 4:** Cephalometric measurements for PWS individuals and control group between 12 and 20 years of age

Measurement		PWS 12–20 years (n = 10)		Control 12–20 years (n = 30)		Difference	t-test *p* value
		Mean	SD	Mean	SD		
Basal sagittal							
n-s-ba	Degree	124.5	6.0	129.1	4.3	− 4.6	0.01
s-n-A	Degree	84.7	2.9	82.4	3.0	+ 2.3	0.05
s-n-B	Degree	81.0	3.0	80.0	2.8	+ 1.0	0.35
A-n-B	Degree	3.7	2.2	2.5	2.2	+ 1.2	0.13
A ḻ n-pg	mm	3.0	2.3	1.4	2.4	+ 1.6	0.08
Basal vertical							
NL/NSL	Degree	5.4	3.8	7.4	1.9	− 2.0	0.02
ML/NSL	Degree	30.7	7.4	28.7	3.8	+ 2.0	0.28
ML/NL	Degree	25.3	7.9	21.3	4.1	+ 4.0	0.05
FA/n-ba	Degree	92.6	4.6	92.2	3.7	+ 0.4	0.78
Anterior face height							
n-sp	mm	49.8	4.6	48.5	3.0	+ 1,3	0.34
sp-gn	mm	67.8	8.7	59.8	4.8	+ 8.0	0.001
n-sp/sp-gn	%	74.3	9.9	81.4	6.4	− 7.1	0.01
Dental							
Ii to A-pg	mm	1.5	2.7	1.5	1.7	0	0.99
ILi/A-pg	Degree	21.8	4.1	23.2	4.4	− 1.4	0.36
ILi/ML	Degree	92.8	7.5	95.1	4.7	− 2.3	0.27
Is to A-pg	mm	6.4	2.6	4.8	2.0	+ 1.6	0.05
ILs/A-pg	Degree	25.7	5.8	23.1	5.6	+ 2.6	0.22
ILs/NSL	Degree	103.9	6.1	102.6	5.7	+ 1.3	0.53
ILi/ILs	Degree	132.5	8.2	132.7	8.4	− 0.2	0.71

Basal sagittal—*n-s-ba*: cranial base inclination, *s-n-A*: maxillary prognathism, *s-n-B:* mandibular prognathism, *A-n-B*: relative prognathism, *A ḻ n-pg*: facial convexity

Basal vertical—*NL/NSL*: maxillary inclination, *ML/NSL*: mandibular inclination, *ML/NL*: mand./max. inclination, *FA/n-ba*: facial axis

Anterior face height—*n-sp*: upper anterior face height, *sp-gn*: lower anterior face height, *n-sp/sp-gn*: ratio

Dental—*Ii to A-pg*: lower incisor protrusion, *ILi/A-pg*: lower incisor inclination to A-pg, *ILi/ML*: lower incisor inclination to ML, *Is to A-pg*: upper incisor protrusion, *ILs/A-pg*: upper incisor inclination to A-pg, *ILs/NSL*: upper incisor inclination to NSL, *ILi/ILs*: interincisor angle

**Table 5:** Cephalometric measurements for PWS individuals and control group over 20 years of age

Measurement		PWS > 20 years (n = 20)		Control > 20 years (n = 60)		Difference	t-test *p* value
		Mean	SD	Mean	SD		
Basal sagittal							
n-s-ba	Degree	129.1	6.1	128.6	3.6	+ 0.5	0.67
s-n-A	Degree	82.5	4.5	83.1	3.8	− 0.6	0.59
s-n-B	Degree	81.4	3.3	80.2	3.9	+ 1.2	0.22
A-n-B	Degree	1.1	3.7	2.9	2.4	− 1.8	0.02
A ḻ n-pg	mm	− 0.2	3.9	1.3	2.9	− 1.5	0.07
Basal vertical							
NL/NSL	Degree	7.5	3.7	8.3	3.2	− 0.8	0.40
ML/NSL	Degree	27.7	5.7	27.4	5.5	+ 0.3	0.81
ML/NL	Degree	20.2	5.2	19.1	5.6	+ 1.1	0.46
FA/n-ba	Degree	96.9	4.6	92.5	4.5	+ 4.4	0.0001
Anterior face height							
n-sp	mm	49.9	3.7	51.1	3.6	− 1.2	0.20
sp-gn	mm	65.5	6.4	64.8	5.5	+ 0.7	0.64
n-sp/sp-gn	%	76.7	7.8	79.3	7.7	− 2.6	0.19
Dental							
Ii to A-pg	mm	1.1	2.6	1.3	2.6	− 0.2	0.89
ILi/A-pg	Degree	20.3	5.8	23.7	5.5	− 3.4	0.02
ILi/ML	Degree	89.7	9.3	95.8	6.9	− 6.1	0.002
Is to A-pg	mm	4.7	2.4	4.3	2.3	+ 0.4	0.59
ILs/A-pg	Degree	23.8	7.3	20.7	5.9	+ 3.1	0.05
ILs/NSL	Degree	106.7	8.5	101.2	6.7	+ 5.5	0.004
ILi/ILs	Degree	135.9	10.7	135.6	10.3	+ 0.3	0.90

Basal sagittal—*n-s-ba*: cranial base inclination, *s-n-A*: maxillary prognathism, *s-n-B*: mandibular prognathism, *A-n-B*: relative prognathism, *A ḻ n-pg*: facial convexity

Basal vertical—*NL/NSL*: maxillary inclination, *ML/NSL*: mandibular inclination, *ML/NL*: mand./max. inclination, *FA/n-ba*: facial axis

Anterior face height—*n-sp*: upper anterior face height, *sp-gn*: lower anterior face height, *n-sp/sp-gn*: ratio

Dental—*Ii to A-pg*: lower incisor protrusion, *ILi/A-pg*: lower incisor inclination to A-pg, *ILi/ML*: lower incisor inclination to ML, *Is to A-pg*: upper incisor protrusion, *ILs/A-pg*: upper incisor inclination to A-pg, *ILs/NSL*: upper incisor inclination to NSL, *ILi/ILs*: interincisor angle

#### Sagittal relationships

A retrognathic mandibula (A-n-B: 5.0°, a difference of + 1.9° from the control group) was found in children with PWS resulting in a mean Class II skeletal pattern (Table 3). They also had a more convex skeletal profile (A ḻ n-pg: 4.3 mm). Young adults (Table 4) showed a reduced cranial base angle (n-s-ba: 124.5°) and a skeletal class II relationship. Adults with PWS (Table 5) had a skeletal Class III relationship (A-n-B: 1.1°, a difference of − 1.8° from the control group).

#### Vertical dimensions

Children with PWS (Table 3) had an increased inclination of the maxilla (NL/NSL: 11.6°, a difference of + 4.4° from the control group) while in young adults (Table 4) this inclination was reduced (NL/NSL: 5.4°, a difference of − 2.0° from the control group). No statistical difference was seen in the adults with PWS (Table 5) compared to the control group.

#### Growth directions

In children with PWS (Table 3), the growth direction axis was posterior (FA/n-ba: 88.2°) while adults (Table 5) had an anterior growth direction (FA/n-ba: 96.9°).

#### Anterior face height

Both upper anterior face height (n-sp: 46.0 mm) and the lower anterior face height (sp-gn: 57.7 mm) were larger in children with PWS (Table 3).

Young adults with PWS (Table 4) showed a longer lower anterior face height (sp-gn: 67.8 mm) and a subsequent smaller n-sp/sp-gn ratio (n-sp/sp-gn: 74.3%) than the control group.

No statistical difference was seen in the adults with PWS (Table 5) compared to the control group.

#### Dental occlusion

Dental cephalometric measurements of children and young adults with PWS were no different to the control group (Tables 3, 4).

Adults with PWS (Table 5) had more proclined upper incisors to the maxillary plane (ILs/NSL: 106.7°, a difference of + 5.5° from the control group) and retroclined lower incisors to the mandibular plane (ILi/ML: 89.7°, a difference of − 6.1° from the control group) and to the A-pg line (ILi/A-pg: 20.3°, a difference of − 3.4° from the control group).

### Comparing all PWS age groups (Tables 6, 7, 8)

**Table 6:** Cephalometric measurements for PWS individuals under 12 years and PWS individuals over 20 years of age

Measurement		PWS < 12 years (n = 12)		PWS > 20 years (n = 20)		Difference	t-test *p* value
		Mean	SD	Mean	SD		
Basal sagittal							
n-s-ba	Degree	129.2	7.0	129.1	6.0	+ 0.1	0.96
s-n-A	Degree	81.1	5.3	82.5	4.5	− 1.4	0.43
s-n-B	Degree	76.1	5.5	81.4	3.3	− 5.3	0.002
A-n-B	Degree	5.0	2.4	1.1	3.7	+ 3.9	0.003
A ḻ n-pg	mm	4.3	2.2	− 0.2	3.9	+ 4.5	0.001
Basal vertical							
NL/NSL	Degree	11.6	4.5	7.5	3.7	+ 4.1	0.06
ML/NSL	Degree	35.9	7.4	27.7	5.7	+ 8.2	0.009
ML/NL	Degree	24.3	7.6	20.2	5.2	+ 4.1	0.001
FA/n-ba	Degree	88.2	5.4	96.9	4.6	− 8.7	0.08
Anterior face height							
n-sp	mm	46.0	4.3	49.9	3.7	− 3.9	0.07
sp-gn	mm	57.7	6.1	65.5	6.4	− 7.8	0.01
n-sp/sp-gn	%	80.1	6.6	76.7	7.7	+ 3.4	0.002
Dental							
Ii to A-pg	mm	1.5	2.3	1.1	2.6	+ 0.4	0.72
ILi/A-pg	Degree	20.7	4.1	20.3	5.8	+ 0.4	0.83
ILi/ML	Degree	93.6	5.4	89.7	9.3	+ 3.9	0.23
Is to A-pg	mm	4.5	3.5	4.7	2.4	− 0.2	0.85
ILs/A-pg	Degree	26.1	8.4	23.8	7.2	+ 2.3	0.44
ILs/NSL	Degree	96.3	7.7	106.7	8.5	− 10.4	0.003
ILi/ILs	Degree	133.2	9.4	135.9	10.7	− 2.7	0.49

Basal sagittal—*n-s-ba*: cranial base inclination, *s-n-A*: maxillary prognathism, *s-n-B*: mandibular prognathism, *A-n-B*: relative prognathism, *A ḻ n-pg*: facial convexity

Basal vertical—*NL/NSL*: maxillary inclination, *ML/NSL*: mandibular inclination, *ML/NL*: mand./max. inclination, *FA/n-ba*: facial axis

Anterior face height—*n-sp*: upper anterior face height, *sp-gn*: lower anterior face height, *n-sp/sp-gn*: ratio

Dental—*Ii to A-pg*: lower incisor protrusion, *ILi/A-pg*: lower incisor inclination to A-pg, *ILi/ML*: lower incisor inclination to ML, *Is to A-pg*: upper incisor protrusion, *ILs/A-pg*: upper incisor inclination to A-pg, *ILs/NSL*: upper incisor inclination to NSL, *ILi/ILs*: interincisor angle

**Table 7:** Cephalometric measurements for PWS individuals between 12 and 20 years of age and PWS individuals over 20 years of age

Measurement		PWS 12–20 years (n = 10)		PWS > 20 years (n = 20)		Difference	t-test *p* value
		Mean	SD	Mean	SD		
Basal sagittal							
n-s-ba	Degree	124.5	6.0	129.1	6.0	− 4.6	0.06
s-n-A	Degree	84.7	2.9	82.5	4.5	+ 2.2	0.17
s-n-B	Degree	81.0	3.0	81.4	3.3	− 0.4	0.74
A-n-B	Degree	3.7	2.2	1.1	3.7	+ 2.6	0.05
A ḻ n-pg	mm	3.0	2.3	− 0.2	3.9	+ 3.2	0.02
Basal vertical							
NL/NSL	Degree	5.4	3.2	7.5	3.7	− 2.1	0.13
ML/NSL	Degree	30.7	7.4	27.7	5.7	+ 3.0	0.23
ML/NL	Degree	25.3	7.9	20.2	5.2	+ 5.1	0.41
FA/n-ba	Degree	92.6	4.6	96.9	4.6	− 4.3	0.02
Anterior face height							
n-sp	mm	49.7	4.6	49.9	3.7	− 0.2	0.91
sp-gn	mm	67.8	8.7	65.5	6.4	+ 2.3	0.42
n-sp/sp-gn	%	74.3	9.9	76.7	7.7	− 2.4	0.46
Dental							
Ii to A-pg	mm	1.5	2.7	1.1	2.6	+ 0.4	0.72
ILi/A-pg	Degree	21.7	4.1	20.3	5.8	+ 1.4	0.47
ILi/ML	Degree	92.8	7.4	89.7	9.3	+ 3.1	0.36
Is to A-pg	mm	6.4	2.6	4.7	2.4	+ 1.7	0.09
ILs/A-pg	Degree	25.7	5.8	23.8	7.2	+ 1.9	0.48
ILs/NSL	Degree	103.9	6.1	106.7	8.5	− 2.8	0.37
ILi/ILs	Degree	132.5	8.2	135.9	10.7	− 3.4	0.39

Basal sagittal—*n-s-ba*: cranial base inclination, *s-n-A*: maxillary prognathism, *s-n-B*: mandibular prognathism, *A-n-B*: relative prognathism, *A ḻ n-pg*: facial convexity

Basal vertical—*NL/NSL*: maxillary inclination, *ML/NSL*: mandibular inclination, *ML/NL*: mand./max. inclination, *FA/n-ba*: facial axis

Anterior face height—*n-sp*: upper anterior face height, *sp-gn*: lower anterior face height, *n-sp/sp-gn*: ratio

Dental—*Ii to A-pg*: lower incisor protrusion, *ILi/A-pg*: lower incisor inclination to A-pg, *ILi/ML*: lower incisor inclination to ML, *Is to A-pg*: upper incisor protrusion, *ILs/A-pg*: upper incisor inclination to A-pg, *ILs/NSL*: upper incisor inclination to NSL, *ILi/ILs*: interincisor angle

**Table 8:** Cephalometric measurements for PWS individuals under 12 years of age and PWS individuals between 12 and 20 years of age

Measurement		PWS < 12 years (n = 12)		PWS 12–20 years (n = 10)		Difference	t-test *p* value
		Mean	SD	Mean	SD		
Basal sagittal							
n-s-ba	Degree	129.2	7.0	124.5	6.0	+ 4.7	0.11
s-n-A	Degree	81.1	5.3	84.7	2.9	− 3.6	0.07
s-n-B	Degree	76.1	5.5	81.0	3.0	− 4.9	0.02
A-n-B	Degree	5.0	2.4	3.7	2.2	+ 1.3	0.20
A ḻ n-pg	mm	4.3	2.2	3.0	2.3	+ 1.3	0.17
Basal vertical							
NL/NSL	Degree	11.6	4.5	5.4	3.2	+ 6.2	0.001
ML/NSL	Degree	35.9	7.4	30.7	7.4	+ 5.2	0.12
ML/NL	Degree	24.3	7.6	25.3	7.9	− 1.0	0.76
FA/n-ba	Degree	88.2	5.4	92.6	4.6	− 4.4	0.06
Anterior face height							
n-sp	mm	46.0	4.3	49.7	4.6	− 3.7	0.06
sp-gn	mm	57.7	6.1	67.8	8.7	− 10.1	0.05
n-sp/sp-gn	%	80.1	6.6	74.3	9.9	+ 5.8	0.11
Dental							
Ii to A-pg	mm	1.5	2.3	1.5	2.7	0	0.98
ILi/A-pg	Degree	20.7	4.1	21.7	4.1	− 1.0	0.58
ILi/ML	Degree	93.6	5.4	92.8	7.4	+ 0.8	0.80
Is to A-pg	mm	4.5	3.5	6.4	2.6	− 1.9	0.19
ILs/A-pg	Degree	26.1	8.4	25.7	5.8	+ 0.4	0.90
ILs/NSL	Degree	96.3	7.7	103.9	6.1	− 7.6	0.025
ILi/ILs	Degree	133.2	9.4	132.5	8.2	+ 0.7	0.87

Basal sagittal—*n-s-ba*: cranial base inclination, *s-n-A*: maxillary prognathism, *s-n-B*: mandibular prognathism, *A-n-B*: relative prognathism, *A ḻ n-pg*: facial convexity

Basal vertical—*NL/NSL*: maxillary inclination, *ML/NSL*: mandibular inclination, *ML/NL*: mand./max. inclination, *FA/n-ba*: facial axis

Anterior face height—*n-sp*: upper anterior face height, *sp-gn*: lower anterior face height, *n-sp/sp-gn*: ratio

Dental—*Ii to A-pg*: lower incisor protrusion, *ILi/A-pg*: lower incisor inclination to A-pg, *ILi/ML*: lower incisor inclination to ML, *Is to A-pg*: upper incisor protrusion, *ILs/A-pg*: upper incisor inclination to A-pg, *ILs/NSL*: upper incisor inclination to NSL, *ILi/ILs*: interincisor angle

#### Sagittal relationships

Young adults (Table 8) had a more prognathic mandibula (s-n-B: 81.0°) than children did (s-n-B: 76.1°), a difference of − 4.9°. Both had a skeletal class II pattern and a convex profile.

Adults (Table 7) showed an even more prognathic mandible than the young adults with a skeletal class III relationship (A-n-B: 1.1° in adults, A-n-B: 3.7° in young adults, a difference of + 2.6°).

#### Vertical dimensions

The inclination of the maxillary plane angle was larger in children (NL/NSL: 11.6°) than young adults (NL/NSL: 5.4°), (Table 8). Adults had reduced inclination of the maxillary and mandibular planes (NL/NSL, ML/NSL and ML/NL) than children.

#### Growth directions

The growth direction axis (FA/n-ba) was more posterior in the young adults and children than in adults (Tables 6, 7, 8).

#### Anterior face height

Both upper anterior face height (n-sp) and lower anterior face height (sp-gn) were significantly smaller in children than in adults (Table 6).

Young adults showed a significant larger lower anterior face height than children (Table 8).

#### Dental occlusion

Upper incisors were more proclined to the maxillary plane in young adults (ILs/NSL: 103.9°) and adults (ILs/NSL: 106.7°) than in children (ILs/NSL: 96.3°), (Tables 6, 7, 8).

## Discussion

Several significant aberrations in the craniofacial morphology in children, young adults and adults with PWS were demonstrated in this study.

The children with PWS showed a retropositioned mandible with skeletal class II relationship, a more convex profile and a posterior growth direction while adults with PWS had a prognathic mandible resulting in skeletal class III relationship, a concave profile with anterior growth direction.

Schaedel et al. [18] using only linear measurements, showed, in a group of 20 individuals with PWS divided in children and adults, that the bony craniofacial structure in both children and adults was smaller than the control group but with a larger posterior cranial base length. In 18 individuals with PWS from 4 to 33 years of age, Belengeanu et al. [19], had similar findings as Schaedel et al. [18] in the linear measurements. The angular measurements showed reduced cranial base angle and the inclination of the mandibula with an anterior growth pattern. The maxilla had a more prognathic position indicating a skeletal class II pattern. A study of 20 children by Giuca et al. [20] confirmed the findings of both Schaedel et al. and Belengeanu et al. [18, 19] in the linear measurements but contrary to Belengeanu et al. [19], no differences were found in the relationship of the jaws to the cranial base.

Because of the difference in age distribution and parameters, it is difficult to compare our results to the three other craniofacial studies of PWS [18–20]. Contrary to Belengeanu et al. and Giuca et al. [19, 20], we found increased lower and upper facial height in children and increased lower face height in young adults.

This study is part of a comprehensive study of oral findings in PWS [10–12]. Saeves et al. [21] investigated the same individuals with PWS in a case-control study regarding severity of tooth wear. Low salivary flow and extensive tooth wear were found to be common findings in PWS with dental wear increasing with age. Tooth wear has an obvious effect on the dentition with reduction of tooth substance. It can also influence the dentoalveolar complex and the facial morphology with reduced lower facial height, smaller gonial angle and relative mandibular prognathism [24, 25]. Krogstad and Dahl [25] explained the alterations in dentoalveolar morphology to increased muscle function. In our study, also increased mandibular prognathism was observed in adults with PWS as compared with the younger age groups, but without changes in the face lower height when compared to the controls.

Tallgren [26], in her study of face height and tooth wear, found that despite advanced tooth wear, dentoalveolar compensation may cause the occlusal vertical dimension to remain constant or even increase. The mechanism that can explain these changes is still not completely understood. Probably there is a morphological adaptation to a change in function [3]. Solow [27] described the adaptability of the dentoalveolar complex to changes in maxilla-mandible relationships with eruptive, alveolar and skeletal compensatory mechanisms. A significant reduction of the vertical measurements with decreased inclination of both the maxilla and mandibula was found in our study comparing adults to children with PWS. Tooth wear can be present since early age in individuals with PWS. This may allow a compensation of the dentoalveolar complex to tooth wear, making reduction of the lower face height difficult to identify, but measurable over time.

Almost all cases in this study were treated with GH, and some even have had a long-term treatment. Long-term treatment with GH appears to improve body composition, reducing body fat, increasing muscle strength and physical function and development progress with height gain in PWS [6, 28]. There is evidence that GH has an effect on growth sites with endochondral ossification such as in the condylar cartilage [29] and it seems possible to improve the facial and mandibular growth with GH treatment resulting in an anterior rotation of the mandible [30] and a more prognathic growth pattern [31]. In our study, linear measurements of the mandible were not included but similar findings are registered: Adults with PWS have a prognathic mandible and a more anterior growth direction compared to the children and young adults. A dentoalveolar compensation of the incisors with retroclined lower incisors and proclination of the upper incisors were also found, probably as a result of this compensation. It is important to be aware of the possible effect on the growth prognosis and changes in the craniofacial morphology in PWS with long-term medication with GH.

This study gives an indication of the craniofacial characteristics for children, young adults and adults with PWS. PWS is a rare condition with large individual variability. Therefore, an individual assessment is essential. Craniofacial morphology is influenced by both genetic and environmental factors. An early diagnosis, careful clinical examination, anamnestic records and making much effort in preparing the patients for dental procedures are important to optimize treatment planning and clinical management in PWS.

## Conclusion

This study may contribute to a better understanding of the craniofacial pattern for children, young adults and adults with PWS and may have a clinical importance when planning dental treatment, such as prosthodontics and/or orthodontics.

It is important to be aware of tooth wear in PWS and to early identify aetiological factors, such as low salivary flow rates and gastro-oesophageal reflux, to prevent pathologic loss of tooth substance and possible changes in the facial morphology.

## Data Availability

All data generated during and/or analysed during the current study are not publicly available because the data includes participants’ personal health information but are available from the corresponding author upon reasonable request.

## Abbreviations

PWS: Prader–Willi syndrome
GH: Growth hormone
SPSS: Statistical Package for the Social Sciences
ICC: Intra-class correlation coefficient
USA: United States of America
